# Getting Through COVID-19: The Pandemic’s Impact on the Psychology of Sustainability, Quality of Life, and the Global Economy – A Systematic Review

**DOI:** 10.3389/fpsyg.2020.585897

**Published:** 2020-11-12

**Authors:** Mogeda El Sayed El Keshky, Sawzan Sadaqa Basyouni, Abeer Mohammad Al Sabban

**Affiliations:** ^1^King Abdulaziz University, Jeddah, Saudi Arabia; ^2^Assiut University, Asyut, Egypt; ^3^Umm Al-Qura University, Mecca, Saudi Arabia

**Keywords:** coronavirus disease, COVID-19, the psychology of sustainability, economic growth, sustainable development, quality of life, world economy

## Abstract

The COVID-19 pandemic may affect the world severely in terms of quality of life, political, environmental, and economic sustainable development, and the global economy. Its impact is attested to by the number of research studies on it. The main aim of this study is to evaluate the impact of COVID-19 on the psychology of sustainability (quality of life), on sustainable development, and on the global economy. A computerized literature search was performed, and journal articles from authentic sources were extracted, including MEDLINE (PubMed), Google Scholar, Science Direct, ProQuest, and Emerald Insight. The references in selected articles were screened to identify any relevant studies. The following inclusion criteria were followed: research articles based on the COVID-19 pandemic, and articles, research papers, journals, and news articles published 2010 to 2020. The exclusion criteria were as follow: psychology research, articles, and journals published before 2010 and research articles having no link with the current pandemic’s impact on the psychology of sustainability, quality of life, and the global economy. Of the initial 350 articles identified, only 61 studies were found to be relevant and meet the inclusion criteria. Based on these articles, the review highlights that compared to developed countries, the developing nations and poor nations such as African countries with compromised health structures have been greatly affected. There are close associations between health, economic, environmental, and political issues globally. The pandemic can be managed if we follow new policies that implement economic and public health changes worldwide. A planned, coordinated approach between the public-private sector is required, designed according to each country’s health system and economy. We can come out of this crisis if we work together and support both developed and developing nations.

## Introduction

There is a high rate of uncertainty related to COVID-19, whose pandemic impacts economic performance, sustainability criteria, and development processes. Haider et al. (2020) mention the effect of coronavirus on health and economic crises. Analysis of its growth in countries, clearly shows that its development leads to crises. Declining GDP rates damage health, education, and industrial progress globally. According to Herbert (2020), COVID-19 affects socio-economic circumstances because of declining global GDP, declining capital flows, fewer investment opportunities, and decreased trading. Not limited to economic loss, this pandemic impacts social parameters like the changes in sustainable psychological development. Globally, the rate of poverty is increasing. The International Monetary Fund (IMF) World Economic Update for June estimated considerable fluctuation in the final ratios (IMF, 2020).

More than 20 million people currently live in extreme poverty; however, Mukhtar (2020) reports that an increase of about 420 million is projected to be living in extreme poverty. Findings gathered by the United Nations Industrial Development Organization (UNIDO) reflect that COVID-19 has resulted in a severe decline in human development for the first time since 1990 (Zandifar and Badrfam, 2020). Their examination reveals that the current global picture lacks socio-economic development. These issues and challenges directly affect an individual’s psychology and assure them of a loss of psychological sustainability and the addition of financial crises. Specifically, with many risks affecting the public, cases of mental crises are increasing (Kang et al., 2019). As a result of people being restricted to their homes and being asked to maintain self-isolation, there is a high chance of someone being severely affected psychologically, which is further impacted by the lack of accurate guidelines or treatment.

When no resources are provided to manage people’s well-being, the situation, including pandemic prevention measures, is reframed and affects psychological health. Concerning the impact on sustainable psychology, Bai et al. (2020) discuss the importance of improved mental health because it affects individual growth and counters restricted personal activities. The authorities’ actions and management criteria for regulating the pandemic are beyond people’s control but adversely impact their exercising, eating habits, gardening, dancing, meditation, learning, and other activities. As a result, people perceive the negative impact on their minds, and the sustainability of their psychological health is damaged (Yao et al., 2020). In the view of Li et al. (2020), COVID-19 impacts the quality of life and mental health as it prejudices human living standards. The joint United Nations Program on HIV/AIDS (UNAIDS, 2020) notes that this pandemic increases the numbers of people suffering from stress and anxiety, conditions that are related to depression. Thus, it is essential to conduct a study to evaluate the impact of COVID-19 from the perspective of quality of life and economic, psychological, and environmental perspectives.

Several research studies have highlighted the severe impact of the COVID-19 pandemic. It is worth noting that the 2013 SARS outbreak experienced in Hong Kong damaged mental health (Fernandes, 2020), but, specific to COVID-19, there are diverse effects on mental health following the imposition of preventive measures. Social distancing, self-isolation, limited meetings, and lack of interaction directly decelerate the economy and mental health. Many countries face declining projected global trade and export volumes. In the view of Allcott et al. (2020), psychological sustainability involves the merger of political perspectives, human development, and economic aspects, and COVID-19 has had an impact on all three. Fetzer et al. (2020) discuss the pandemic’s impact on the global economy as self-isolation results in loss of business revenue. Restrictions on consumers being able to purchase ultimately result in an economic downturn. Apart from this, stresses are being constantly imposed on people worldwide that negatively affect their minds and decrease economic activity (Iacus et al., 2020). COVID-19’s considerable impact has emotionally traumatized individuals; the handling of the situation has reduced their level of comfort, socially, economically, and environmentally, according to Cartwright et al. (2020). The amalgamation of these factors triggers a high level of stress in people’s minds, which, meanwhile, affects economic development, as it ruins efforts for developmental projects (Pirouz et al., 2020).

We have faced several epidemics in the past. Asian nations were impacted by the Middle East Respiratory Syndrome (MERS outbreak), and West Africa was under the attack by the Ebola virus. They also influenced the socio-economic equilibrium, affected public health, and caused numerous deaths similar to what we are facing with COVID-19 (Marin, 2018; Lawanson and Evans, 2019). The pandemic has affected all types of businesses. There are shortages of medical equipment such as masks and Personal Protection Equipment (PPE), etc. It has made us realize how fragile our systems are and that no country can face this crisis on its own. A targeted and collaborative approach is required.

The main aim of this current research is to evaluate the impact of the COVID-19 pandemic on the sustainability of the quality of life, i.e., how people are tending toward stress, anxiety, depression, and other health/mental issues. Not limited to this alone, the study discusses the pandemic’s impact on sustainable development psychologically and economically. Given that changes in psychological sustainability link with people’s living style, and how they deal with their life situations, there is a need to conduct a study in this direction. Currently, there are reports and research articles that separately discuss the impact of COVID-19’s rapid spread on the health system, mental health, sustainability, and the global economy (Allcott et al., 2020; Banerjee, 2020; Pirouz et al., 2020). Research related to the pandemic’s simultaneous effects on the psychological, economic, and environmental paradigms is required. That is why this study explores information about and human experiences that influence their quality of life psychologically, economically, and environmentally.

Due to the pandemic, trade has also been largely affected. The impact of this chaos will have a long-term effect on globalization. All private and public sectors are under its influence (Donald, 2020). Previously, all large companies had goals focused only on financial gain. However, now the level of interconnected trade has lost its meaning. There has been an unequal distribution of the benefits associated with globalization (Roome, 2011). The more powerful governments and those who own major conglomerations should realize that unless we work together, the overall quality of life will be compromised globally. Working culture and environment and the worker’s s policies should be looked into to obtain a flexible, innovative, and empathetic workplace for everyone to deal with this crisis.

Therefore, the aim of this research report is to:

• Analyze the impact of the COVID-19 pandemic on the sustainability of quality of life
• Determine the effect of the COVID-19 on the economic, social, and political factors relating to the sustainable development environment
• Evaluate the impact of the COVID-19 pandemic on the global economy
• Examine the organizational changes and solutions for dealing with the COVID-19 pandemic, and
• Highlight the effect of support of the world trade environmental infrastructure in tackling the condition of COVID-19.

## Methodology

### Study Design

All the guidelines and principles were followed while preparing the methodology for this research. A thorough literature search was conducted, and after proper evaluation and analysis, relevant literature was identified and included for the present review.

To accomplish the desired objectives, all the studies related to the topic published from Jan 2010 to June 2020 were selected. It was assumed that including some publications of the previous decade would be helpful in reflecting upon the practices and strategies that were implemented in situations previously like the global economic recession A computerized literature search was performed and journal articles from authentic sources were extracted, including MEDLINE (PubMed), Google Scholar, Science Direct, ProQuest, and Emerald Insight. The References in the selected articles were screened to identify any relevant studies. The literature search was performed by including the following keywords: “Coronavirus” OR “pandemic” OR “SARS-CoV-2” OR “COVID-19” OR “sustainability” OR “quality of life” OR “Global economy” OR “psychology and Organizational changes and COVID-19.”

### Inclusion and Exclusion Criteria

**FIGURE 1:**
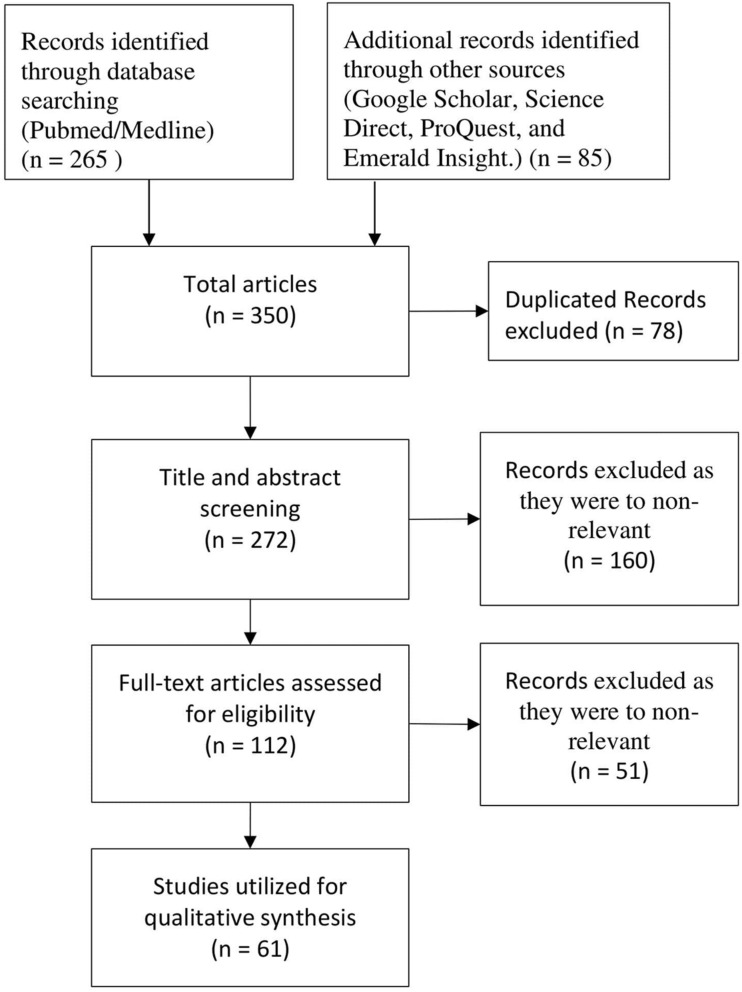
Flow diagram illustrating the literature search and selection criteria (according to PRISMA, Preferred Reporting Items for Systematic Reviews and Meta-Analysis; Moher et al., 2009).

The inclusion criteria were: research articles based on the COVID-19 pandemic and articles, research papers, journals, and news articles published from 2010 to 2020; articles on sustainability management related to virus pandemic. The exclusion criteria were: psychology research, articles, and journals published before 2010; research articles having no link with the current pandemic’s impact on the psychology of sustainability, quality of life, and the global economy and additionally, articles that were in languages other than English. The process of retrieving and screening the studies according to these criteria in this systematic review is shown in Figure 1. After an initial search, a total of 265 articles were identified in MEDLINE (PubMed), and 85 through other databases. After removing the duplicate records, 272 titles and abstracts were screened. Finally, only 61 studies were found to be relevant and meet the inclusion criteria.

## Literature Review

### Psychology of Sustainability

According to Chandler (2020), the psychology of sustainability and the criteria for relative development are associated with socio-economic progress, leading to improved living standards. The study by Srivastava et al. (2020) proposed that the management of sustainability relates to ecology, equity, and economy. Meanwhile, Cartwright et al. (2020) observed that COVID-19 affects the quality of life as overall economic, ecological, and equity conditions have changed. According to Bastola et al. (2020), psychological and sustainability factors contribute to well-being and allow psychological development. Recycling, dismantling, and demolishing factors are affected by sustainability. The psychology of sustainability also relates to deconstruction, recoverability, and oxygenation (Iacus et al., 2020). Using the micro-dimension of awareness creates an opportunity to increase awareness that ultimately enables involvement in self-centered development criteria.

### Impact of Coronavirus Disease on the Psychology of Sustainability and Quality of Life

Political, environmental, and economic aspects collectively determine sustainable development psychologically (Bowen et al., 2017). These aspects also determine how humans should spend their current lives so that the same quality of life can be transferred to and enjoyed by future generations. According to Garfin et al. (2020), minimal changes in human life slowly alter the future, but future generations will not perceive their lives as the outcome of change; they will think that people before them have led their lives in similar ways. World Wars and pandemics have impacted our present lives. Moreover, the COVID-19 pandemic has dramatically impacted the political, environmental, and economic aspects of human life on which psychological development and sustainability are dependent. This ultimately affects the quality of life by disturbing people’s living standards. The outbreak of COVID-19 in early 2020 has agitated social problems and threatened the economies of the world (Nicola et al., 2020). According to Arden and Chilcot (2020), growth and development in different countries have stopped. The financial stability of both developed and undeveloped countries has been shattered as the pandemic has targeted many lives. Human beings are highly dependent on socialization. Because social distancing and lockdown are necessary precautions for avoiding COVID-19, the resultant increased stress and depression directly lessens the quality of life (Balasubramanian, 2020). Most people around the globe are losing their jobs, i.e., their income. Profit margins and revenue generated by various organizations have dropped. To save the economy and their citizens’ psychological health from the pandemic, many countries have developed strategies, but years of struggle will be required to regain economic stability.

Moreover, the COVID-19 pandemic and the associated crises have traumatized people’s psychological well-being, especially employees who have lost their jobs. The well-being of employees working from home is compromised as the gap between their professional and personal lives has reduced (Pratt and Frost, 2020). The situation has stressed employees and has led to an uncomfortable and aggressive relationship with the organization, which has directly, or indirectly ruined their psychological sustainability on a macro level. According to Mahase (2020), the world before and after this pandemic will never be the same as people have isolated themselves, millions of lives have already gone, and the global economy has slowed exponentially. COVID-19 has imposed the harsh realities of unemployment, illness, and bereavement on people, and long-lasting hardships and struggles are required to mitigate the situation’s negative impact. Coronavirus has substantially impacted people’s psychology and has created an extensive psychological experiment on human beings, which will eventually change the overall lifestyle of current and future generations.

Knowing that the condition of the current pandemic has diverse effects on psychological sustainability, disturbs the quality of life, and restricts people to having to deal with preventive measures. However, according to Di Fabio (2017), the management of the psychology of sustainability helps to foster well-being and enhance working conditions within society. There are changes in behavior in which most people are suffering from stress, anxiety, and fatigue (Wang et al., 2020). The full lockdown restriction of staying in the home negatively impacts on human living standards. Professional examination reveals that increasing cases related to post-traumatic stress, nutritional deficiencies, and psychological issues have been reported. Oher psychological impacts of COVID-19 include a growing fear of leaving home.

### Impact of Coronavirus Disease on Sustainable Development

#### Impact of Coronavirus Disease on Economic Conditions

The interdependency of overall prosperity and integrity of health emphasizes human dependence on the state of the economy. Econometric analysis of the world’s economic growth rate shows that the current pandemic has led to widespread health crises and economic damage. According to Haider et al. (2020), the economic situation depends on the GDP rate helping to affect economic recovery measures. Global economic crises due to COVID-19 reveal economic decline. Moreover, the report by Allcott et al. (2020) highlights the declining economy related to fluctuations in GDP rates. In the current scenario, the GDP rate has shrunk by approximately 4.2%, the first time for a pandemic (Fetzer et al., 2020). Another report estimated that a difference of 7% is projected in the coming period if the same conditions continue (IMF Blog, 2020). Additionally, there will be a considerable number of further crises if the IMF faces losses. The overall rates of advanced economies like those of Europe and America have declined the same as emerging economies (Kang et al., 2019).

COVID-19 impacts global trade and investment. In Fernandes’ view (2020), changing global trading volumes can be observed, and all industries are eventually affected by the pandemic. The World Trade Organization (WTO) supports this view (WTO.org, 2020). Its graphs represent the changes in the average value of trade, which includes a contribution by the IMF to change the economic outlook, which, in turn, relates to growing global poverty and declining living standards (Bastola et al., 2020). GDP growth projections make it apparent that living standards are heading to extreme poverty at an increased rate. Subsequently, this negatively affects the economy, as it leads to economic crises.

Chandler (2020) reports that COVID-19 has impacted labor by 68% in just 2 weeks, which signifies the loss of many jobs and directly increases stress levels. The highest unemployment rates are in Asia, Europe, and America. In Asia and the Pacific regions, unemployment rates have headed toward a decrease of 4.5%. In America, Europe, and Central Asia, it is 10.5%. Apart from this, fluctuations in trading sectors have led to a decline in manufacturing, textile, cosmetics, and many more industries. According to Iacus et al. (2020), COVID-19 has enormously impacted the income ratios in developed and developing countries. A shift in fiscal policy packages has been assessed across 162 countries. It was distributed as Austria (∼17.80% of GDP), France (∼15.30% of GDP), Qatar (∼13.00% of GDP), United States (∼10.50% of GDP), and Australia (∼9.70% of GDP). Monetary stimulus packages across these 162 countries were distributed as Bahrain (26% of GDP), China (14.14% of GDP), Germany (12.49% of GDP), and the United Kingdom 9.09% of GDP; Sarkodie and Owusu, 2020).

#### Impact of Coronavirus Disease on Environmental Issues

Along with the impact of COVID-19 on economic conditions, there is an environmental impact, which Cartwright et al. (2020) discuss. The COVID-19 situation impacts global emissions, especially in relation to the release of emissions into the atmosphere. The lessening of CO_2_ emissions relates to the effect of COVID-19 on human development, which in the view of Bastola et al. (2020), led to the 2020 financial crisis. The restrictions on human development and declining rates of change evolved along with the loss of environmental degradation. It was observed that in Asian and European countries, the extent of the ambient particulate matter has declined significantly (Gautam and Trivedi, 2020; Kasha, 2020). Urban and industrial areas have less carbon monoxide and aerosol in the present situation (Gautam and Trivedi, 2020; Holthaus, 2020). These environmental gains mirror the losses in the fields of health, education, income, and trade (Bai et al., 2020).

#### Impact of Coronavirus Disease on Political Issues

The coronavirus’s impact can be observed politically when lockdown and self-isolation result in reduced export and import rates. In the current situation, it is difficult to carry out activities designed to run the economic wheel (Haider et al., 2020). Furthermore, there has been a considerable shift in the policies and strategies related to economic policies. On the industrial level, there has been a decline in line with industrial restriction. Even though support is being provided for business opportunities to deal with the COVID-19 pandemic, there are still restrictions on mobilizing current development. According to Bastola et al. (2020), political parties have provided funds and highlighted financial support to ensure people’s survival during COVID-19. Moreover, the impact of lockdown, isolation, and rescheduling, as well as the spread and fear of the virus, have resulted in new political perceptions.

### Impact of Coronavirus Disease on the Global Economy

According to Moti and Ter Goon (2020), global economic integration is required to deal with the implications of the coronavirus pandemic. A balanced partnership between the public-private sector, which takes into account the contextual economy and health system, and is specific to each country’s situation, will help national as well as international health and economic recovery. The world’s total GDP depends on the economies of separate countries; China’s economy is the largest contributing economy, and the United States’s, the second largest. Due to COVID-19, most factories are moving toward closure and stopping the production of goods. This lack of production of goods and services has a great impact on the consumers, and no significant purchasing practices have been recorded since the spread of COVID-19. In the same context, declining sales are forcing the international market to face the situation. Brands like Apple, Toyota, Jaguar, Land Rover, and many more are facing the loss of investors and consumers (Ahmad et al., 2020). According to Qiu et al. (2020), Hyundai has shut down its business services and supply operations due to a lack of consumer purchases. Starbucks has shut outlets as consumers cannot purchase. A reduction in the import rate of oil by China resulted in a decline in international oil prices. Multiple uncertainties have been observed in the consumption of smartphones as both demand and supplies are worsening. The car production company, S&P, has faced declining purchases (Ahmad et al., 2020). The aftermath of COVID-19 will thus impact the global economy. According to an IMF report (IMF, 2020), the issues related to the current pandemic will restrict the global economy. Overall sustainable development criteria are expected to collapse. The distribution of economic policy globally in response to COVID-19 was conducted across 166 countries. It demonstrated that the United States is at the top, followed by Sweden. There are few countries with no economic policy such as Kazakhstan, Ukraine, Yemen, Liberia, and Denmark (Sarkodie and Owusu, 2020).

### Organizational Survival Envisioned by Human Resources During the Pandemic

During the outbreak of the coronavirus, management styles need to be changed to tackle operations and reduce the chances of a crisis, according to Ågerfalk et al. (2020). This includes consideration of online management, as online networks can help organizations survive. In Donald’s view (2020), there are two major types of organizational arrangements: traditional and a new post-pandemic system. The traditional structure (pre-pandemic model) has existed during the past five or six decades. However, its demerits were clearly evident during this pandemic. It lacks clarity when defining roles and responsibilities. There is a disparity in outcome attainment, and system working-conditions are less efficient. Although power allocation is part of the matrix structure, in a crisis, it can lead to instability and loss of control (Roome, 2011), resulting in an inadequate organizational structure. The new-age model (post-pandemic) focuses on the innovation, knowledge, and better required skillsets (Guan and Huang, 2014). Organizations that are quickly adaptive build creativity and possess a sharing attitude will be in a better situation to manage its employees post-pandemic. The old models based on power and control need to shift to a more open, flexible, and modernized culture.

In the current situation, typical hierarchical organizations will not result in better outcomes as the approach to allocate power and authority to a specific group will restrict working conditions. Human resources (HR) has envisioned a new model, using distributed leadership, innovation, and continuous training to adapt to the changing times (Hu, 2014) to obtain effective results. According to McConnell’s (2020) study, organizations with networked, hierarchical, distributed leadership styles, cross-training practices, and flexible guidelines using survival techniques to tackle COVID-19 conditions.

### Support of World Trade Environmental Infrastructure to Tackle COVID-19

The impact of COVID-19 is not limited to sustainable development, as there are challenges associated with global trade management. According to the United Nations Environment Program (United Nations Environment Programme [UNEP], 2020), trade is essential for saving livelihoods and increasing economic cooperation. Whether it is related to COVID-19 or typical situations, trade infrastructure boosts the confidence level of operations and allows improvement of the transparency of environmental trade infrastructure (Deshmukh and Haleem, 2020). Other than this, multiple actions and procedures need to be followed for the management of the COVID-19 situation (Hishan et al., 2020). With support from world trade’s environment infrastructure, there is an opportunity to supply food and health products. This may help organizations to avoid unnecessary export and import practices. Development in world trade’s environmental infrastructure helps ensure public interest and government support to effectively analyze the development choices. Gilbert et al. (2020) confirm the importance of world trade environmental infrastructure to boost the confidence level and increase the transparency of economies. Deshmukh and Haleem (2020) consider that the transparency of shared strong data or collections of information contributes to supporting the managed infrastructure required for COVID-19.

When West Africa suffered from a massive outbreak of the Ebola virus, it led to a high death rate and affected the country at numerous levels (Smith et al., 2019). Socio-economic disparity, slow growth rates, shortage of food, and loss of businesses and jobs resulted. We are facing a similar situation presently, which demands that the health, economic, and environmental policies should be modified so we can recover from this crisis and collaborate in the future efficiently (Smith et al., 2019).

## Research Findings

The analysis helped to evaluate the impact of COVID-19 on the psychology of sustainability, quality of life, and the global economy. In the initial search, we found 350 articles, including 78 duplicate articles. After title and abstract screening, we were left with 272 articles. Of these, 112 were assessed for eligibility, and only 61 met the inclusion criteria. After extraction, the relevant articles were categorized into the following subheadings to provide a clear description: the author and year of the published article; the assessment of the key findings due to COVID-19; the present implications caused by the pandemic, and future perspective that will help in the recovery from this crisis situation. See Table 1. The focus of this work is to review the research work published specifically in response to COVID-19. It is interesting to observe that the majority of the studies were from 2019 to 2020. This is justified as the pandemic occurred in very recent times. Hence the research mainly highlighted its current impact globally and the lessons learned from current scenarios. Among these, the majority are review articles, and only a few of them were randomized and controlled clinical trials, which assessed the economic, environmental, health, and sustainability impacts.

**TABLE 1:** Impact of COVID-19 and future perspective to improve health, economy, sustainability, and quality of life.

Author/Year	Assessment	Implications	Future perspective
Moti and Ter Goon, 2020	Impact on the health and the economy	As compared to the developed countries, the developing nations and poor nations such as African countries with a compromised health structure have been greatly affected.	New policies should be implemented that focus on economic recovery as there has been inflation for essential goods and services. Government should strategically execute revised norms to combat the pandemic. It should implement a mitigation policy and a post-pandemic policy. The mitigation policy should target the nation’s health sector. It will include various new changes to deal with the pandemic such as defined containment measures, protection of health workers with additional benefits. Increased supply of the sanitizers and other Personal Protection Equipment (PPE). Regional opening of testing centers. Online education aimed at improving the awareness regarding the risks associated with COVID-19 and how to successfully manage them. The post-pandemic recovery policy will ensure that individual follow social distancing properly and also abide by the lockdown rules. For people to sustain they can be allowed to work for specific hours at offices, avoiding gatherings, etc. so that the businesses as well as the citizens are not at a loss.
Sarkodie and Owusu, 2020	Assessed impact on the environment, health and economy.	Air pollution has declined, however, the amount of medical waste has dramatically increased. Several fiscal measures, changes in the monetary policies and economy recovery have been shared by private sectors across numerous countries. Of 143 countries, United Kingdom ranks with the highest level of uncertainty in the assessment of pandemic uncertainty. Among 162 countries, United States has implemented the greatest policy cuts.	Due to the crisis, many developing and developed countries will face recession as they have introduced several new policies such as fiscal, and monetary measures, and additional welfare costs with health policies. This has impacted developing and weaker nations badly. To deal with the economic slowdown, these countries will adapt to a scaled effect. Priority will be given to resource depletion over the sustainable utilization Governments across all the nations should aim to achieve an outcome ensuring that health, economy and sustainable development are not compromised once we recover from the pandemic.
Berchin and de Andrade Guerra, 2020	Impact on sustainable development of all the sectors	COVID-19 has led to an increased demand of healthier and organic food making the various food systems susceptible. The impact of the pandemic is largely observed among women, children, elderly, wage workers, and small and medium enterprises (SMEs).	Several measures should be taken to achieve a balance among all sectors: Regional mobilization: Policies to ensure trade is continued and involves private firms to help small and medium enterprises. Global support to combat the risk associated with the cross-border transactions. Enhanced accessibility of technology: With the changing world and innovation occurring at a faster pace, technology should be provided in all the rural areas so people can access all kinds of information post-pandemic. Revised Policy: Policies to strengthen the overall system and not only focus solely on financial growth. Education: Encourage use of technologies and distance learning irrespective of the region or nationality.
Donald, 2020	Impact on management and leaders; business and the decision models	The design models of many organizations were based on the lack of creativity and flexibility, which were favorable 30 to 40 years ago. The importance of decision making, risk management, and involvement of stakeholders that worked previously needs modification in the present situation.	New organizational structures are required, based on innovation, confidence, risk taking attitude, and flexibility. In the newly-designed organizations there should be no restricted roles, responsibilities, or structure. Training should be conducted so everyone is adaptive and can modify their needs as per requirements.
Kottika et al., 2020	Impact on SMEs involving business and consumer markets	There has been economic breakdown in the growth of United States GDP as it decreased by 4.8% in the first quarter of 2020. In European nations, GDP shrunk by 3.8% in the same period	It is important to develop entrepreneurial personality traits as it is clearly shown that their attitude plays a significant role in the orientation of SMEs. A high quality of service should be provided to consumers following specific protocols that are open to change depending on the market situation.
Etemad, 2020	Impact on the quality of life, economy, as well as the organization of various institutions.	There has been a slowdown in the functioning of all sectors whether in a large or a small city, rural area or urban place. However, SMEs are the ones that have been impacted the most due to the pandemic.	The situation prior to the crisis should be assessed closely, and processes followed regarding entrepreneurial and internationalization perspectives. A review and reevaluation of sectors should be done to maximize financial support to those who cannot recover on their own.

COVID-19 caused greater chaos than previous pandemics. It is represented in all the studies: the global spread, its implications, and how it has ruined all sectors, small and large. Even though the assessment of various factors has been done in the studies, clear, conclusive steps to be followed are not included in many of them. The research has shown that developing countries are in a far worse situation in managing their health systems and economy than developed countries. Additionally, people of the rural areas, the elderly, women, and children are undergoing major stresses and life changes due to the pandemic. Even though air pollution has decreased tremendously in all countries, post-pandemic there will be piles of medical waste, which will impact the entire environment. These research projects show that the management and leadership systems based on power and control are not a sustainable option in the future. Long use of a lack of creativity, technological usage, and strict policies cannot be continued now.

Table 1 clearly shows that the pandemic has disrupted the balance among all the nations. Though the impact is mainly seen in the health sector and the economy, on a deeper level, everything is affected. There is the struggle of SMEs, social distancing norms, working from home, the new era of online teaching, the suffering of daily-wage workers, a crisis situation for the restaurants, hotels, and aviation department, changes in fiscal and monetary policies, the psychological impact on health professionals and health workers, and the extra workload on the sanitation department. In terms of analyzing the research findings of the included studies, it is very clear that the impact of the pandemic has been assessed by one or more of the factors (health, economy, environment, sustainability, or management). However, it is clear that a conclusive result based on the psychology of sustainability, overall well-being, and global economic implications is lacking. Thus, this has contributed to the goal of the present study: how can we learn from these challenges faced by humans globally?

## Discussion

Analysis of the impact of COVID-19 on the global economy highlights that various elements affect economic conditions. The study by Pirouz et al. (2020) observes that the current pandemic hurts GDP as it directly weakens a region’s overall economy. This is supported by the view that the loss of consumer consumption affects the economy of regional economies, too (Fetzer et al., 2020). In other words, multiple socio-economic factors lessen the economic rate and decelerate the global economy.

The collected data also discusses the impact of COVID-19 on psychological sustainability. The information gathered revealed that humans experience an increasing rate of uncertainty when stress, anxiety, and depression are continually increasing (UNAIDS, 2020). According to Zandifar and Badrfam (2020), there are various ways by which COVID-19 can affect sustainable development psychologically. Evaluation of elements related to sustainability reveals that associations of social, environmental, and economic factors lead to psychological sustainability practices. The collected data reveals the impact of COVID-19 on the psychology of sustainability. The current situation has a consistent impact on people’s mind-sets. As a result, there is a need to adapt services to tackle mental health issues to allow people to survive with an improved quality of life (Li et al., 2020). With the declining economy, the contribution of COVID-19 can be observed in the global emission system. Labor can expect to decline in the future, and there is a high chance of an individual facing job loss. This overall situation leads to stress and restricts people in developing the economy sustainably (Chandler, 2020). These employment issues are also linked with psychological factors as they are the leading cause of stress and depression, and ultimately hurt the quality of life (Banerjee, 2020).

The collected data shows that HR departments are now changing working criteria and focusing on alternative working solutions for organizations. Allcott et al. (2020) observe that COVID-19 forces HR to shift management from close-ended to open-ended leadership styles. A dispersed workforce and its interdependency on loose criteria are considered necessary for organizational survival in the pandemic. Apart from this, HR prefers to adopt flexible guidelines and cross-training practices to provide practices and services to manage the pandemic’s result. These sorts of instructions and guidelines help HR ensure the survival of an organization and save corporations from the calamities experienced by COVID-19 (Fernandes, 2020).

Similarly, most organizations are reacting in a managed manner to increase their productive outcomes. It has been clear that a declining projection rate is observed globally, and no improvement for the projected rates is expected (Pratt and Frost, 2020). According to a report published by WHO (WTO.org, 2020), a loss of capital flows and a decline in annual charges is decelerating economic conditions. Furthermore, there are also considerable COVID-19 effects in the form of losses faced by global trade and investment practices. Analysis of the impact of COVID-19 on the economy reveals that the world’s economy is expected to face further decreases in volumes and global trade projections because the current situation is worsening day by day.

Along with this, there is an impact of COVID-19 politically, which results in reduced exports and imports. Politically, a high level of funds is required to support a country’s regulation (Bowen et al., 2017). The spread of the virus endangers the overall sustainability of development. The situation during COVID-19 has been managed by the support of trade’s environmental infrastructure as various macro-level elements help to ensure sustainable development. With the help of improved access to advanced technologies, it is anticipated that production processes can make development more efficient (Haider et al., 2020). There is a shortage in the supply of drugs and medicine for mental health issues. This, according to pharmacists, is a significant issue. It hinders the development of health services and makes it difficult for practitioners to improve their patients’ quality of life (UNAIDS, 2020). There is an opportunity for support of world trade environmental infrastructure as this allows corporations to work with advanced infrastructures and increased interest levels. Moreover, world trade support systems may enhance the efficient supply of food and medicines. With this, there is a clear avoidance of import and export practices without advanced infrastructures (Herbert, 2020). With infrastructure support, there is a high chance of transparency in the management of economies when these companies support the developed infrastructure (Arden and Chilcot, 2020). World trade environmental infrastructure support includes a focus on planting trees and promoting sustainable practices to provide useful opportunities to increase healthy regional recovery.

The impact of COVID-19 on psychological sustainability can be examined by observing the changes in people’s behavior. Di Fabio (2017) shared the thoughts of people quitting the workplace because of the pandemic, as this also affected educational institutions. Along with this, a lack of healthcare-related facilities contributed to the negative psychological impact on sustainability. Balkhi et al. (2020) found that various psychological factors affect people’s behavior, ultimately changing global lifestyles. More than 80% of people worldwide show more concern about safety as they prefer to reduce physical contact with others (Wang et al., 2020). Around 23% of people face extremely anxious conditions due to mental well-being (UNAIDS, 2020). Other than this, the peoples’ behavioral changes include increasing exhaustion and fatigue that directly restrict them from working toward development.

There is a link between the psychology of sustainability, sustainable development, and economic crises because all these depend on the quality of life and related improvement (Rothan and Byrareddy, 2020). Zenker and Kock (2020) mention that the COVID-19 pandemic changes people’s lifestyles globally by affecting their social, economic, and environmental contexts. Multiple reasons drag the psychology of sustainability toward losses in profit margins and revenue generated. Kang et al. (2019) highlighted the crisis rate of COVID-19 by reflecting on job crises, consumption rates by consumers, and increasing unemployment rates. One COVID-19 impact on individual well-being is that compromised work policies make it difficult for employees to survive peacefully. In the same context, multiple changes are observed in the situation experienced globally as the condition of self-isolation makes people less interested in harming the economy. Some of the destructive realities associated with coronavirus disease include illness, unemployment, bereavement, long-lasting hardship, and struggle in handling the situation (Fetzer et al., 2020). Other than this, there are diverse effects of COVID-19 economically, socially, and environmentally. Rothan and Byrareddy (2020) used a survey to explore the psychological impact of the ongoing pandemic and found that people suffer from a lack of confidence and from the infection itself. There is less concern about the maintenance of health, as most people do not have access to infection control measures. Along with this, there is no realization of the situation’s gravity. People can only understand it in terms of their situations.

Multiple uncertainties result from the outbreak of COVID-19, as this affects the individual and humanity at large. COVID-19 has an impact on the psychology of sustainability, the nature of thought, and the attitudes expressed. Within this context, continual stresses are imposed on people from restrictions that negatively affect their minds. Along with this, COVID-19 stresses employees and others and creates an uncomfortable relationship with a peaceful life, directly and indirectly affecting psychological sustainability.

People’s psychologies have changed, as they are dependent on the global situation, currently negatively affected by COVID-19. The result is that the COVID-19 pandemic and associated crises have traumatized people’s psychological well-being by disturbing their social, economic, and environmental peace. The lack of work policies leads to stress, as criteria for managing the situation are, as yet, undeveloped. The data involving regional GDP rates, economic efficiencies, sales rates, and trade rates reveal an economic impact of COVID-19. All these aspects are directly and indirectly associated with the pandemic. A fall of 7.2% in GDP has been observed in economies.

However, we can learn from past crises to survive the present global economic loss. In the Greek financial breakdown, 700,000 jobs were lost between 2008 and 2014 (KEPE, 2015). More than 35% of medium-sized businesses were largely impacted as compared to small businesses. Entrepreneurs managed to come out of the crisis by providing us some key findings that can help us to better deal with COVID-19. They ensured that their products met all their consumers’ needs, lowered their prices (Bourletidis and Triantafyllopoulos, 2014), utilized advanced tools and technologies to provide something meaningful (Giannacourou et al., 2015). Additionally, entrepreneurial or managerial personality traits played a significant role in defining company successes (Elenurm et al., 2014; Espíritu-Olmos and Sastre-Castillo, 2015).

The various findings reveal that fluctuations regarding trading system volumes when this relates to losses or improvements in industries worldwide. They also reveal an increased proportion of living standards worldwide is heading toward extreme poverty. Crises are facing energy production as there are lower CO_2_ emissions, which mirrors the fact that human development and progress are declining. Changes in management styles are required to deal with how society operates and reduce the chance of further crises. However, the adoption of a networked, hierarchical, distributed leadership style, cross-training practices, and flexible guidelines will benefit corporations in tackling the COVID-19 crises. Finally, the impact of COVID-19 can be tackled by the support of the world trade environmental infrastructure, which is known to boost the confidence levels of corporations in operations and improve the transparency of global trade.

## Implications and Future Perspectives

Humans have witnessed several previous crises in different regions and countries, and humanity emerged from them. We should think about creating a global change to prevent further suffering caused by the COVID crisis. It is highly probable that a recurrence of the present crisis will strike the global population more severely. It is therefore desirable that:

1. Government be proactive and implement planned precautions now before the situation worsens.
2. Management, institutions, and organizations develop their skill sets demonstrate sustainability, resilience, and innovation as COVID-19 has compromised traditional business management and systems.
3. Globally, nations plan and sanction policies for the collective good instead of their own self-interests.
4. All educational institutions provide necessary guidance and professional help to deepen understanding of crisis management. This will help individuals to become aware, protect themselves, and avoid any risks or harm caused by their negligence.
5. All the important health organizations, health professionals, scientists, and researchers be provided a targeted fund that provides sufficient training and understanding regarding the pandemic’s impact on public health, so the next crisis is dealt with more effectively.
6. Humanity take a collective approach to avoid unnecessary harm to the environment. The earth can be saved if each one of us becomes more responsible, provides support, and care for one another.

We can learn a lot from this pandemic and become more capable of dealing with any future crisis.

## Conclusion

This pandemic has taught us that the entire world is connected. If we do not work together and cooperate, humanity will suffer drastically. We need to implement a few changes so that we not only emerge from this crisis but are also able to continue with our lives in a healthy and sustainable way. Firstly, it is important that marketing processes are modified. Instead of blindly following brands and getting attracted to the logos, etc., companies should try to meet their customers’ requirements. There should be a shift in business approaches from financial targets to what is best for customers. Unnecessary use of plastic products should be strictly prohibited. Secondly, any health-related issue should be handled at a global level. If we limit ourselves to our own specific race, ethnicity, culture, nationality, and background, humanity will not be able to sustain this pandemic. A broader view and understanding of public health need to be the key drivers for all political parties. Thirdly, globally oriented with specific goals, WHO should be the decision-maker for our health and well-being. Guaranteed budgets should be allocated, and policies prioritizing health in different regions and cultures should be planned. Fourthly, people from all cultures and interests, such as health professionals, scientists, environmentalists, researchers, politicians, sociologists, and ethicists should cooperate and work to improve the current situation. Additionally, all the policies related to the functioning of society, taxation, fiscal policy, environmental issues, economy, and health should be changed so humans can survive on planet Earth harmoniously.

## Author Contributions

All authors have made a substantial, direct and intellectual contribution to the work, and approved it for publication.

## Conflict of Interest

The authors declare that the research was conducted in the absence of any commercial or financial relationships that could be construed as a potential conflict of interest.
