# Corrigendum: Getting Through COVID-19: The Pandemic's Impact on the Psychology of Sustainability, Quality of Life, and the Global Economy – A Systematic Review

**DOI:** 10.3389/fpsyg.2021.700815

**Published:** 2021-05-26

**Authors:** Mogeda El Sayed El Keshky, Sawzan Sadaqa Basyouni, Abeer Mohammad Al Sabban

**Affiliations:** ^1^King Abdulaziz University, Jeddah, Saudi Arabia; ^2^Assiut University, Asyut, Egypt; ^3^Umm Al-Qura University, Mecca, Saudi Arabia

**Keywords:** coronavirus disease, COVID-19, the psychology of sustainability, economic growth, sustainable development, quality of life, world economy

In the original article, we neglected to include the funder. The Deanship of Scientific Research at Umm Al-Qura University, Grant Code: 18-HUM-1-03-0002, to SB. The updated funding statement is shown below.

## Funding

The authors would like to thank The Deanship of Scientific Research at Umm Al-Qura University for supporting this work by Grant Code: 18-HUM-1-03-0002.

The authors apologize for this error and state that this does not change the scientific conclusions of the article in any way. The original article has been updated.

